# Aberrant gesture use in autism spectrum disorders is unrelated to motor abnormalities

**DOI:** 10.1007/s00406-025-02093-x

**Published:** 2025-08-22

**Authors:** Anastasia Pavlidou, Avram Tolev, Daniela Reinhold, Gerrit Steinberg, Thomas J. Müller, Sebastian Walther

**Affiliations:** 1 Translational Research Center, University Hospital of Psychiatry and Psychotherapy Bern, University of Bern, Bolligenstr. 111, 3000 Bern 60, Switzerland; 2 Department of Psychosomatic Medicine, University of Basel and University Hospital Basel, Basel, Switzerland; 3 Privatklinik Meiringen AG Willigen, Meiringen, Switzerland; 4 Department of Psychiatry, Psychosomatics, and Psychotherapy, Center of Mental Health, University Hospital of Würzburg, Würzburg, Germany

**Keywords:** Gestures, Nonverbal communication, Social cognition, Motor impairments, Manual dexterity

## Abstract

**Background:**

Gesture deficits are well-documented in youth with autism spectrum disorder (ASD), yet research in adults with ASD remains limited. Understanding the nature of gesture deficits in adulthood is essential for identifying their underlying mechanisms and potential impact on communication and daily functioning. The aim of the current study examines gesture performance in adults with ASD to explore whether these deficits persist beyond childhood and how they relate to motor impairments.

**Methods:**

We included 19 patients diagnosed with ASD and 19 age-and-gender matched controls. Gesture performance accuracy was assessed in both groups using the Test of Upper Limb Apraxia (TULIA) which was subjectively rated according to the manual by an independent single rater who was blinded to the group allocations, while manual dexterity was assessed using the performance-based coin-rotation task. We further assessed motor impairments in patients using standardized well-established motor scales to examine their potential contributions to gesture accuracy.

**Results:**

Individuals with ASD exhibited significant gesture deficits compared to controls, while manual dexterity remained preserved. Tool-based gestures appeared to be the most affected. Though ASD individuals exhibited numerous motor impairments they were not associated with gesture deficits.

**Conclusions:**

Our findings suggest that gesture deficits in ASD are not driven by the presence of motor impairments. However, given the small sample size, these results should be interpreted with caution. Future studies with larger and more diverse samples are needed to further investigate the mechanisms contributing to gesture difficulties in ASD.

## Introduction

Autism spectrum disorder (ASD) is a complex neurodevelopmental condition characterized by persistent difficulties in social communication and interaction, along with restricted, repetitive patterns of behavior, interests, or activities, as defined in the Diagnostic and Statistical Manual of Mental Disorders, Fifth Edition (DSM-5; [1]). Globally, ASD is estimated to affect approximately 1:100 children with prevalence estimates growing over time [2].

Abnormal motor behaviors are one of the earliest impairments observed in children with ASD with many demonstrating consistent impairments in several aspects of motor control and execution [3, 4]. Several studies focusing on motor abnormalities report significant deficits in fine and gross motor control, such as manual dexterity, gait, as well as object and postural control in both children and adolescents with ASD [5–10]. Beside these basic motor deficits, children/adolescents with ASD also display deficits in executing skilled gestures [11–14]. Gestures are visible bodily movements used alone or in combination with language to facilitate social communication [15]. They are fundamental in increasing understanding among interacting individuals and serve as predictors in the development of language skills in children [16, 17].

Impairments in imitation of skilled gestures have been particularly emphasized in children/adolescents with ASD and are thought to be related to dysfunction of the mirror neuron system and is a core feature of ASD [18–25]. However, research examining performance accuracy of skilled gestures in children/adolescents with ASD, which included not only imitation of gestures that are visually presented to them but also execution of skilled gestures following verbal command, report an overall deficit in gesture performance beyond imitation compared to typically developed children [19, 26]. In addition, observational studies have noted that children/adolescents with ASD tend to use gestures less frequently than typically developed children during social communication with peers, family members and caregivers [27–31].

Even though co-speech gesture use and timing differences are well-documented in adults with ASD [32, 33], and comprehensive reviews confirm deficits in deictic and emblematic gestures [34], no prior study has examined skilled gesture performance using a well-established test that examines gesture ability in the context of motor planning and motor execution, and how they relate to motor abnormalities in adults with ASD. At the same time, some of the early motor deficits should have been compensated by maturation and training [35–37]. Thus, adults with ASD may have less motor impairments and therefore perform better when gesturing. To this end, the current study aimed to assess gesture performance accuracy in adults with ASD using the well-established Test of Upper Limb Apraxia (TULIA; [38]) due to its strong psychometric properties, including high internal consistency and interrater reliability, as well as the availability of normative data across clinical such as schizophrenia and depression and non-clinical populations [38–42]. TULIA assesses gesture performance across two domains (imitation and pantomime) and three gesture categories (meaningless, intransitive, and transitive), capturing deficits in both cognitive and motor dimensions. It evaluates content (semantic accuracy or structural correctness), spatial configuration (precision of limb positioning and movement trajectory), and timing (fluency and sequencing), where temporal disruptions such as hesitations or fragmentation can reduce scores due to their impact on overall gesture ability. In addition, TULIA can be administered within approximately 10 min with minimal materials, and requires only limited verbal instructions making it particularly suitable for use in clinical populations such as ASD. Importantly, TULIA distinguishes between gesture ability and gesture use, allowing us to isolate motor-cognitive gesture impairments from social-communicative limitations. In addition to the TULIA test, we used several well-established standardized motor scales together with a performance-based fine motor skill task [43] commonly used in Parkinson’s, schizophrenia, and depression research to test fine and gross motor abilities and evaluate their relationship with gesture abilities in adults with ASD. Using these well-established standardized tests will allow us to contextualize our findings within existing research highlighting commonalities and distinctions across disorders. We further explored whether TULIA performance relates to spontaneous gesturing or self-reported symptoms such as depression and negative symptoms to test whether gesture deficits reflect reduced expressive behavior and/or broader affective or motivational disturbances. We hypothesize that adults with ASD will have difficulty in performing gestures accurately, particularly when imitating gestures, and that these deficits will be associated motor abnormalities and higher depression and negative symptoms.

## Methods

### Participants

Individuals with ASD were approached at the specialized outpatient clinic of the University Hospital of Psychiatry and Psychotherapy, Bern, Switzerland. We enrolled 19 subjects with ASD. In addition, we selected the records of 19 individuals who participated as healthy controls in past and ongoing projects on gestures [40, 44]. Handedness for all participants was assessed using the Edinburgh Handedness Inventory [45]. Patients were matched 1:1 with controls from an existing database using a blinded dataset visible only for age, gender and handedness to prevent selection bias. Matching was within ± 1 year for age, and both groups were balanced for gender and handedness. Written informed consent was obtained from all participants and the study was approved by the local ethics committee in the canton of Bern (BASEC 2017-01145), and thus complies with the tenets of the declaration of Helsinki. Autism spectrum disorders were diagnosed following extensive interviews with patients and their families, including questionnaires, rating scales and tests as detailed below.

### Autism diagnostic tools

All participants included in the ASD group had a formal clinical diagnosis according to DSM-5 criteria, made by qualified clinicians [1]. These assessments were based on multidisciplinary evaluations, which typically include developmental history interviews, cognitive testing, and clinical judgment. In addition, we used the Autism Diagnostic Observation Schedule, Second Edition (ADOS-2) Module 4, which is specifically designed for adolescences and adults suspected of having ASD [46] as an accompanying standardized measure to characterize symptom presentation at the time of testing, but it was not used as the sole basis for diagnostic inclusion. The ADOS-2 Module 4 consists of semi-structured tasks, as well as conversational prompts that provoke specific behaviours relevant to ASD, including difficulties with reciprocal social interaction, atypical communication patterns, and the presence of restricted or repetitive behaviours. Within this framework, gesture use is assessed primarily through two scored items: one evaluating descriptive or instrumental gestures (e.g., pointing, indicating), and another assessing emphatic gestures that convey affect or emphasis. These items provide a broad clinical impression of the spontaneous use of gestures but do not isolate or systematically quantify gestural ability. Examples of tasks include open-ended conversations, storytelling, and structured activities designed to provide opportunities for the observation of social and communicative behaviours. Administration and scoring were conducted by trained and certified clinicians to ensure reliability and adherence to standardized protocols [46].

### Cognitive-affective tasks

We used two validated theory of mind instruments to assess patients’ ability to infer the mental states and emotions of other people. The first is the Reading the Mind in the Eyes Test (RMET; [47]), which uses 36 greyscale photographs of the eye region of different faces, where each is paired with four mental descriptors or emotions (i.e. “confident,” “nervous,” “playful,” “skeptical”). Patients are instructed to pick the best description that matches what is being conveyed by the eyes. The score ranges from 0 to 36 with higher scores indicative of better theory of mind abilities. The second is the Movie for the Assessment of Social Cognition (MASC; [48]) which assesses theory of mind in a naturalistic and context-rich manner as it involves watching a short film depicting social interactions among four characters. Participants are then asked to answer 45 multiple-choice questions about the characters' thoughts, feelings, and intentions. Each question provides four response options, reflecting correct mental state inference, under-mentalizing (reduced complexity in understanding mental states), over-mentalizing (excessive or inaccurate complexity), or unrelated/incorrect inferences. Scoring is based on the number of correct responses, with a total score ranging from 0 to 45. Higher scores indicate better theory of mind abilities.

Furthermore, we used two self-report questionnaires to assess autistic traits and empathy from patients’ subjective perspective. The first is the Autism Spectrum Quotient (AQ; [49]), which measures the extent of autistic traits in patients. It consists of 50 items divided into five subdomains: social skill, attention switching, attention to detail, communication, and imagination. Participants respond to each item on a 4-point Likert scale (1 = “definitely agree” to 4 = “definitely disagree”). Scoring is dichotomized, with responses coded as 0 or 1 depending on alignment with autistic traits, with the total score ranging from 0 to 50. Higher scores indicate more autistic traits are present. The second is the The Cambridge Behavioural Scale Empathy Quotient (CBS-EQ; [50]), and it is designed to assess empathy. It consists of 60 items, 40 of which are scored, and 20 filler items. Responses are rated on a 4-point Likert scale (1 = “strongly agree” to 4 = “strongly disagree”). The total score ranges from 0 to 80 with higher scores indicative of greater empathy. Scores are often categorized as follows: below 30 (low empathy), 30–50 (average empathy), and above 50 (high empathy).

### Gesture performance task

The accuracy of hand gesture performance was investigated using the Test of Upper Limb Apraxia (TULIA; [38]). The TULIA tests gestures in two domains, i.e. imitation (gesture performance following demonstration by the examiner) and pantomime (gesture performance following verbal command). Within each of these domains, three semantic categories of gestures exist: meaningless novel gestures (e.g. place the hand flat on your head), intransitive symbolic gestures (e.g. waving good bye), or transitive tool related gestures (e.g. use a hammer). Participant and examiner face each other while sitting on a chair with both their hands flat on a table. Participants perform the gestures with their dominant hand, while the experimenter demonstrates the gestures in a mirrored manner. The experimenter (A.T) performing the gestures is the same for all patients with ASD and was trained prior to the start of the study to reduce variability and ensure uniform delivery. Performance of the TULIA is video-recorded and subsequently rated subjectively by an independent examiner who is blind to the groups, using the TULIA manual by Vanbellingen et al. [38]. One rater (S.W.) was responsible for all videos to reduce bias and was trained by the original developers of the task. Scores per item range 0–5, with 5 resembling perfect accuracy and 0 unrecognizable movement. Ratings take errors in timing, trajectory or spatial configuration into account, as well as severe content errors, such as body-part as object errors. The TULIA total score ranges 0–240, with a cut-off of 194 for patients with apraxia, while any performance scoring below 210 is classified as a gesture deficit [41].

### Motor assessments

Manual dexterity in both groups was measured with the coin rotation task. In this task participants rotate a 0.50 SFr coin (1 cm diameter) between the thumb, index and middle finger of their dominant hand for 10 s as fast as possible. The performance is repeated three times and video recorded for evaluation. The number of half-turns is calculated and corrected for coin drops using the following formula: Coin Rotation Score = half turns—[(coin drops × 0.1) × half turns]. The mean of the second and third trial was used for further analysis [42, 51].

Further, in patients we also assessed motor abnormalities using well-established standardized motor scales such as: the Bush Francis Catatonia Rating Scale (BFCRS; [52]) where a score of 1 in two or more items of the first 14 items of the scale signifies catatonia [52]; the Unified Parkinson’s Disease Rating Scale motor part (UPDRS-III; [53]), where a score of 2 or more on key motor items (i.e. tremor and rigidity) or a score of 3 on one of the other items signifies parkinsonism [54]; the Abnormal Involuntary Movement Scale (AIMS; [55]) where a score of at least 2 or more in two items, or a score of 3 or more in one item fits the criteria for dyskinesia [56]; and finally the Neurological Evaluation Scale (NES; [57]) for neurological soft signs.

### Self-report symptom severity

 ASD participants also completed the Self-Evaluation of Negative Symptoms (SNS; [57]) to assess negative symptoms and the Beck Depression Inventory (BDI; [58]) to assess depression severity.

### Statistical analyses

All statistical analyses were done using R Studio (version 2024.12.0 + 467) The Shapiro–Wilk tests indicated that TULIA and coin rotation performance scores were normally distributed, while for the motor scales only UPDRS III and NES followed a normal distribution. Demographic characteristics, as well as coin rotation and TULIA total performance scores were compared between groups using t-test. To further assess between group differences in TULIA across domains and categories we used linear mixed-models. Group (patients and controls), Domain (Imitation and Pantomime), and Category (Meaningless, Intransitive, and Transitive), as well as their interactions were added as fixed effects, while a single random intercept estimated for each participant was added as random effects to account for repeated measures. Post-hoc tests were conducted to evaluate significant differences between groups using Bonferroni correction since our TULIA scores were normally distributed. Finally, TULIA scores, manual dexterity and motor-rating scales using nonparametric Spearman rank correlations to evaluate any association between gesture performance and motor behaviors. In an exploratory analysis we also examined TULIA gesture performance associations with the gesture items of ADOS-2 (Descriptive and Emphatic), as well as self-reported negative and depressive symptoms. We used the false discovery rate to correct for multiple comparisons since not all scales followed a normal distribution. P-values < 0.05 were considered significant.

## Results

### Demographic and clinical characteristics

Each group included 12 men and 7 women. Groups did not differ in age, handedness, and manual dexterity but did in hand gesture performance with patients performing less accurately than controls. According to the TULIA cut-off scores (< 194), 63% of the patients qualified for apraxia (Table 1). At the time of testing, 30% of the patients acquired at least a high-school diploma and completed vocational training after graduating from basic school, 25% worked in regular employment, while 75% depended on some form of financial aid from either the social security systems or from their families.

**Table 1:** Demographic and clinical characteristics

	ASD (n = 19)	HC (n = 19)	t-value; p-value
*Demographics*			
Age	37.4 ± 2.5	38.9 ± 2.7	− 0.3; 0.7
EHI	68.3 ± 12.5	81.3 ± 6.0	− 0.9; 0.4
*Task*			
CR	14.2 ± 0.5	14.4 ± 0.7	− 0.3; 0.8
TULIA total	190.6 ± 3.4	218.3 ± 3.0	− 6.1; < 0.0001*
*Psychopathology*			
Stereotyped behaviors_ADOS-2_	0.7 ± 0.2	/	/
Overall communication_ADOS-2_	4.5 ± 0.4	/	/
Overall Social_ADOS-2_	8.1 ± 0.4	/	/
Communication + social_ADOS-2_	12.5 ± 0.7	/	/
Descriptive gestures_ADOS-2_	1.21 ± 0.2	/	/
Emphatic gestures_ADOS-2_	1.42 ± 0.2	/	/
RMET	23.3 ± 1.0	/	/
MASC	31.6 ± 1.5	/	/
AQ	33.2 ± 2.1	/	/
CBS	23.8 ± 2.8	/	/
BDI	11.2 ± 2.3	/	/
SNS	21.3 ± 1.3	/	/
*Motor scales*			
AIMS	3.8 ± 0.9	/	/
BFCRS	2.5 ± 0.3	/	/
NES total	7.2 ± 0.9	/	/
UPDRS III	5.7 ± 0.8	/	/

Values represent the mean ± SEM for each group

*ADOS-2* Autism Diagnostic Observation Scale 2nd Edition; *AIMS* Abnormal Involuntary Movement Scale; *ASD* Autism Spectrum Disorder; *AQ* Autism Spectrum Quotient; *BDI* Becks Depression Inventory; *BFCRS* Bush Francis Catatonia Rating Scale; *CBS* Cambridge Behavioral Scale; *CR* Coin Rotation; *EHI* Edinburgh Handedness Inventory; *HC* Healthy Controls; *MASC* Movie for the Assessment of Social Cognition; *NES* Neurological Evaluation Scale; *RMET* Reading the Mind in the Eyes; *SNS* Self-Evaluation of Negative Symptoms; *TULIA* Test of Upper Limb Apraxia; *UPDRS* Unified Parkinson’s Disease Rating Scale

*Denotes a significant difference between groups

All patients met the criteria for ASD in accordance to the ADOS-2 Module 4 demonstrating a range of severity across its domains (Table 1; [46]). Of the 19 patients, 10 (52.6%) displayed restricted or repetitive behaviors characterized as mild, while 9 (47.4%) showed none. This is not surprising and aligns with previous research that indicate that restricted or repetitive behavior typically declines in frequency and visibility with age and many adults with ASD learn to hide or control such behaviors [58–60]. In addition, 12 patients (63.2%) displayed mild to moderate deficits during reciprocal communication, while 7 (36.8%) exhibited more severe communication deficits, such as significant difficulties in initiating or sustaining conversations. Moreover, 8 patients (42.1%) showed mild impairments in social reciprocity, such as eye contact or facial expression use, while 11 patients (57.9%) exhibited severe social difficulties, including challenges in emotional engagement and understanding social norms. The combined score capturing the overall severity of social and communicative challenges suggests that 13 patients (68.4%) displayed significant deficits in both. Specific to the gesture items assessed in ADOS-2, 3 patients (15.8%) exhibited a typical gesture profile while the majority of patients showed either mild abnormality (42.1%) or a clear, clinically significant deficit (42.1%) for the Descriptive gesture item. In contrast, the Emphatic gesture item showed a greater overall degree of atypicality. Only 2 patients (10.5%) displayed typical gestures in this category, whereas 6 patients (31.6%) exhibited mild abnormality, and the majority of patients (57.9%) displayed a clinically significant deficit (Table 1).

Only 6 patients (32%) reported to be taking some form of medication at the time of testing, with 4 taking antidepressants (two patients were on Serotonin-Norepinephrine Reuptake Inhibitors—SNRI’s; one patient was on Serotonin-Antagonist Reuptake Inhibitors—SARI’s; and finally one patient was on Selective Serotonin Reuptake Inhibitors—SSRI’s) and 2 taking antipsychotics. In addition, 15 (79%) patients had comorbid lifetime psychiatric disorders with 7 (37%) having depression, 4 (21%) having both depression and attention deficit hyperactivity disorder, 1 (5%) having depression with cannabis abuse, 1 (5%) having depression with childhood epilepsy, 1 (5%) with attention deficit hyperactivity disorder and finally 1 (5%) with dyslexia.

Notably, the majority of patients exhibited intact theory of mind abilities, with only six patients (31.5%) showing challenges on the RMET (score < 22; [47] and just one patient (5%) experiencing difficulties on the MASC (Table 1; [48]). However, we should acknowledge that these observations reflect task-specific performances and may not capture subtle or context-sensitive theory of mind challenges that may arise from everyday social interactions and thus do not imply generalizable theory of mind functioning. In contrast, according to self-reports, 16 patients (84%) scored above 26 on the AQ [49], reflecting the presence of autistic traits, while 14 patients (74%) scored below 30 on the CBS-EQ [50], indicating generally low levels of empathy for the majority of ASD patients (Table 1).

All patients exhibited a number of motor abnormalities in accordance to the motor rating scales (Table 1), with the majority of patients scoring above the cut-off scores for catatonia and parkinsonism (73%), as well as dyskinesia (63%). In addition, patients on average had minimal self-reported depressive symptoms in the BDI scale (n = 15 minimal; n = 2 mild; n = 1 moderate, and n = 1 severe). Furthermore, all patients self-reported experiencing moderate to severe negative symptoms, particularly in the domains of social withdrawal, alogia, and avolition (Table 1).

### Gesture performance

**Fig. 1:**
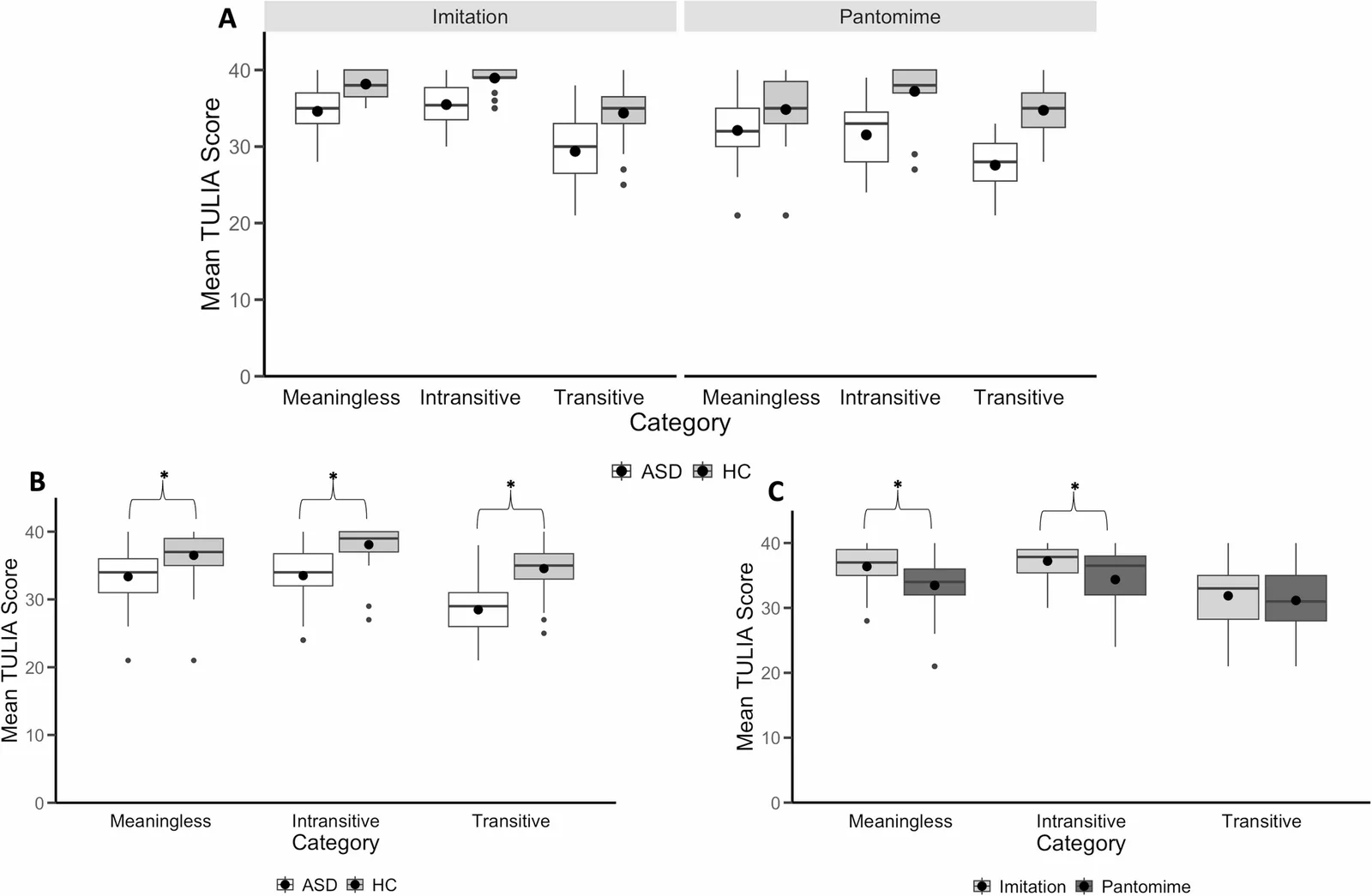
**A**–**C** Results. **A** Box plot comparing gesture performance scores between ASD patients (white) and healthy controls (grey) across both domains (imitation and pantomime) and three categories (meaningless, intransitive and transitive). Post-hoc comparisons were not computed as the three-way interaction was not significant. **B** Box plot comparing gesture performance scores between ASD patients (white) and healthy controls (grey) across categories irrespective of domain. Post-hoc pairwise comparisons showed that ASD patients performed worse in all categories, with the transitive gestures being the most impaired. **C** Box plot comparing gesture performance scores between Imitation (beige) and Pantomime (dark grey) across categories irrespective of the group. Post-hoc pairwise comparisons showed that both groups performed worse in the pantomime meaningless and intransitive categories but not transitive, where their performance was similar across domains. For each box plot the upper and lower bound of each box represents the 75th and 25th percentiles of the distribution, whereas the top and bottom ends of the whiskers represent the 95th and 5th percentiles of the distribution, respectively. The median is represented by the thick horizontal line inside the box and the mean by the black dot. *Denotes a significant difference between groups or domains (p < 0.05)

ASD patients performed worse in both domains and all categories compared to controls (Fig. 1A–C). Linear mixed models revealed a significant main effect of Group, Domain, and Category (all F > 28.8; all p < 0.0001). Though the three-way (F = 1.5; p = 0.2; Fig. 1A) and Group × Domain (F = 2.2; p = 0.1) interaction was not significant, the Group × Category (F = 4.4; p = 0.01; Fig. 1B) and Domain x Category (F = 3.2, p = 0.04; Fig. 1C) interactions were. Post-hoc comparisons revealed that patients performed worse in all categories (all p < 0.01; Fig. 1B) particularly in the transitive category (p < 0.001; Fig. 1B) compared to controls. Both groups performed worse in the transitive category (p < 0.01) compared to the intransitive and meaningless category. Moreover, both groups performed worse in the pantomime domain for the meaningless and intransitive categories (all p = 0.0001; Fig. 1C) but not for transitive (p = 0.3; Fig. 1C), where performance was similar across domains.

### Associations of gesture performance

#### Psychopathology

We observed no associations between TULIA performance and spontaneous gestures as measured in the ADOS-2 or with self-reported affective and motivation disturbances (all rho < 0.4, all p > 0.4; Table 2).

**Table 2:** ASD correlations for TULIA with psychopathology and motor

	TULIA-rho	p-value
*Psychopathology*		
Descriptive gestures_ADOS-2_	− 0.3	0.5
Emphatic gestures_ADOS-2_	0.1	0.7
BDI	0.4	0.4
SNS	0.2	0.5
*Motor*		
AIMS	0.1	0.7
BFCRS	−0.1	0.8
NES total	0.4	0.4
UPDRS III	0.3	0.5
CR	−0.3	0.5

*ADOS-2* Autism Diagnostic Observation Scale 2nd Edition; *AIMS* Abnormal Involuntary Movement Scale; *BDI* Becks Depression Inventory; *BFCRS* Bush Francis Catatonia Rating Scale; *CR* Coin Rotation; *NES* Neurological Evaluation Scale; *SNS* Self-Evaluation of Negative Symptoms; *TULIA* Test of Upper Limb Apraxia; *UPDRS* Unified Parkinson’s Disease Rating Scale

#### Motor domains

Likewise, no associations between TULIA total score with any of the motor-rating scales or with the manual dexterity score were observed (all rho < 0.4, all p > 0.4; Table 2).

## Discussion

Here, we aimed to test gesture performance and manual dexterity in adults with ASD and matched healthy controls. We hypothesized worse gesture performance for ASD patients, especially in the imitation domain, and that these deficits will be linked to manual dexterity and motor impairments. As expected, ASD patients demonstrated severe impairments in gesture performance accuracy across all semantic categories, with transitive (tool-based) gestures being particularly affected. Interestingly, these deficits were present irrespective of the domain. However, the triple interaction of group-by-domain-by-category was not significant. While we noted pronounced motor abnormalities in ASD patients, manual dexterity appears to be preserved. Surprisingly, the severity of motor abnormalities was unrelated to the observed gesture impairments in ASD patients.

The observed impairments in gesture performance accuracy among adult patients with ASD in the current study, are consistent with multiple studies involving children/adolescents suggesting that gesture performance deficits continue well into adulthood for ASD [11, 12, 25]. Notably, these deficits are evident not only during imitation but also during the performance of pantomime gestures, which aligns with recent findings proposing that individuals with ASD have a generalized impairment in gesture performance that extends beyond imitation-specific difficulties [19, 26]. These overall deficits in hand gesture performance are similar to those we observed in patients suffering from schizophrenia and depression in our previous studies using the TULIA [39, 40, 61].

While deficits were evident in each category, tool-based gestures (transitive) seemed to be the most affected in ASD individuals. Correct production of tool-based gestures relies on sensorimotor integration, complex motor planning, and high-order cognitive processes requiring precise coordination between action and perception, often involving learned motor representations associated with tool use [62]. Thus, the pronounced deficits in tool-based gestures in the current study may be closely linked to disruptions in the neural mechanisms supporting action observation and execution [63–65]. In fact, there is evidence pointing to reduced corticospinal excitability during the observation of tool-based hand gestures compared to static within the putative mirror neuron system particularly in premotor areas and inferior frontal gyrus in adults with ASD [66]. Interestingly, our previous studies on schizophrenia and depression also revealed specific gesture deficits, which differed from those observed in our current ASD sample. Specifically, patients with schizophrenia exhibited more pronounced impairments in pantomime meaningless gestures, which are novel gestures and the only category in the TULIA assessment independent of prior gesture knowledge, semantic properties, or motor representations [41]. These deficits were also associated with reduced activation and connectivity in key regions of the brain's praxis network, including the supplementary motor area, inferior frontal gyrus, and inferior parietal lobe [67, 68]. In contrast, patients with depression displayed deficits across all gesture types except for pantomime meaningless gestures [39]. These findings suggest distinct underlying mechanisms contributing to gesture impairments in these disorders.

Beyond gesture deficits, motor impairments also play a distinct role in these disorders. In our current ASD sample, motor impairments were evident despite preserved manual dexterity, yet they appeared to be unrelated to gesture deficits [11]. Previous research in children/adolescents with ASD has shown that gesture deficits persist even after controlling for basic motor skills and postural knowledge, suggesting that these impairments cannot be solely attributed to deficits in motor abilities or gesture knowledge [69]. In contrast, in schizophrenia, motor, postural knowledge and manual dexterity deficits were closely linked to gesture deficits, suggesting a shared underlying mechanism [40, 61]. Meanwhile, in depression, motor, postural knowledge and manual dexterity deficits were present but, similar to ASD, did not show a direct relationship with gesture impairments [39]. This suggests that for both ASD and depression, the gesture deficits are more likely driven by cognitive or social-communicative factors rather than motor dysfunction. In fact, in our depression sample working memory highly correlated with gesture performance [39], while visual attention rather than motor skills appeared to be a contributing factor in ASD according to Gowen et al. [70]. Unfortunately, our current study did not assess cognitive deficits, which limits our ability to explore their potential contribution to gesture impairments in our ASD sample. Additionally, the absence of deficits in theory of mind, which have been proposed to play a role [19], makes it difficult to determine whether impairments in basic hand gesture performance contributes to social-communicative impairments in our ASD sample. Exploratory analyses found no significant associations between TULIA gesture performance abilities and spontaneous gesturing during the ADOS-2, nor with self-reported depression or negative symptoms. This suggests that TULIA is more equipped to capture specific deficits in the planning and execution of imitative and pantomime gestures, which makes it more reliable for detecting impairments in these domains. In contrast, spontaneous gesturing can mask these limitations, as individuals might adapt or compensate in real-time, making such impairments less apparent. Similarly, the absence of associations with self-reported symptoms implies that basic gestural deficits may not directly reflect subjective affective or motivational disturbances. In addition, skewed distributions further limited meaningful statistical interpretation for our self-reported questionnaires. Further research is needed to disentangle the specific cognitive and social communicative processes underlying these deficits.

While our findings indicate clear gesture and motor deficits in the ASD sample, the small sample size (N = 19) presents several limitations. First, the TULIA relies on subjective scoring, which may introduce some variability despite the structured rating system and demonstrated inter-rater reliability [38]. Second, the limited number of participants reduces the generalizability of our results, making it uncertain whether these deficits extend to the broader ASD population. Additionally, the small sample size, although reliably detected gesture deficits, lowers statistical power, increasing the risk of failing to detect potential associations between gesture performance, and motor deficits [71, 72]. Individual differences in symptom severity, and comorbid conditions may also have a disproportionate influence on the findings, making it difficult to distinguish relevant patterns from incidental variability in gesture performance ability. Although our data suggest that gesture deficits in ASD are unrelated to motor impairments these conclusions must be interpreted with caution. Future studies with larger ASD samples are necessary to confirm these findings and further investigate the cognitive, social-communicative and neural mechanisms underlying gesture impairments in ASD. Further, although gesture and social-cognitive difficulties have been observed in other clinical populations such as schizophrenia and depression our present focus is solely on autism. The comparisons were included only to highlight the need for diagnosis-specific investigations into gesture performance, as underlying mechanisms may differ.

Overall, our findings suggest that individuals with ASD exhibit distinct gesture deficits that appear to be unrelated to motor impairments, with tool-based gestures being the most affected. These patterns of results differ from that observed in schizophrenia and depression suggesting distinct mechanisms across these disorders in relation to gesture deficits. Further investigation with larger sample sizes is needed to determine whether these gesture deficits in ASD stem from differences in motor execution, social cognition, or underlying neurofunctional mechanisms (e.g. motor planning network or social-cognitive network), and how they interact with broader communicative and cognitive challenges compared to schizophrenia and depression.

## Declarations

### Data availability

Participants did not provide consent to sharing their data.
